# Survival analysis of under-five mortality using Cox and frailty models in Ethiopia

**DOI:** 10.1186/s41043-017-0103-3

**Published:** 2017-06-02

**Authors:** Dawit G. Ayele, Temesgen T. Zewotir, Hemry Mwambi

**Affiliations:** 1 Department of Epidemiology, Bloomberg School of Public Health, The Johns Hopkins University, 615 North Wolfe Street, Baltimore, MD 21205 USA; 2 School of Mathematics, Statistics and Computer Science, University of KwaZulu-Natal, Pietermaritzburg, Private Bag X01, Scottsville, 3209 South Africa

**Keywords:** Hazards, Interaction effect, EDHS, Frailty, Under-five mortality

## Abstract

**Background:**

The risk of a child dying before reaching 5 years of age is highest in sub-Saharan African countries. But in Ethiopia, under-five mortality rates have shown a substantial decline.

**Methods:**

For this study, the Cox regression model for fixed and time-dependent explanatory variables was studied for under-five mortality in Ethiopia. We adapted survival analysis using the Cox regression model with 2011 Ethiopian Demographic and Health Survey data.

**Results:**

From the results, it was found that under-five children who live in Addis Ababa had a lower hazard (risk) of death (*p* value = 0.048). This could be as a result of higher health facilities and living standards in Addis Ababa, compared to other regions. Under-five children who lived in rural areas had a higher hazard (risk) of death compared to those living in urban areas. In addition, under-five children who lived in rural areas had 18% (*p* value = 0.01) more hazard (risk) of death than those living in urban areas. Furthermore, with older mothers, the chance of a child dying before reaching the age of 5 is lower.

**Conclusion:**

The chances of a child dying before reaching the age of 5 are less if the mother does not become pregnant again before the child reaches the age of 5. Therefore, giving birth when older and not becoming pregnant again before the child reaches the age of 5 is one means of reducing under-five mortality.

## Background

In sub-Saharan African countries, under-five mortality is higher when compared to other countries and eight times higher than WHO European Region [1, 2]. Even though different measures are taken to reduce under-five mortality, under-five mortality is still higher (including Ethiopia) and the rate is above 100 deaths per 1000 live births [3, 4].

Based on the 2000, 2005, and 2011 Ethiopian Demographic and Health Surveys, the risk of under-five mortality shows decline over time. This decline is due to the progressive and consistent implementation of the health interventions since 1960 [5–8]. But the risk is till high in Ethiopia. In addition to under-five mortality, trends of crude death rates also show decreases [9–12].

The pattern of under-five mortality is known to have increasing trend. However, the trends of under-five mortality rate at global level showed decreasing pattern by 53%,. This is the estimated rate of 91 deaths per 1000 live births in 1990 to 43 deaths per 1000 live births in 2015. Based on the records from WHO, average annual rate of reduction for under-five mortality is from 1.8% a year over the period 1990-2000 to 3.9% for 2000-2015. This reduction is not sufficient to meet the MDG 4. [28]. Having this in mind, using survival analysis for lifetime distributions is one of the main difficulties in the investigation of life data. Survival analysis can be used to determine life potentiality presumptions in different times. Comparing the lifetime distributions of various investigated groups [13, 14] is one of the main problems in the analysis of life data. In most cases, the impact of explanatory variables on the lifetime as the dependent variable is modelled using regression models. These models are an important part in survival analysis [15, 16]. Therefore, this kind of model is known as the Cox regression model or the proportional hazards regression model [17]. It is advantageous to use the Cox regression model because it has both nonparametric and parametric parts at the same time: the parametric part being due to the parameter *β* in the model. But the distribution of failure time is assumed known. It is nonparametric in the sense that *λ*_0_ is an unspecified function in the form of the baseline undefined hazard function. Therefore, this part of the model is the nonparametric part. Because of this characteristic, the model is more flexible as measurements are always done with error involved. When the response time is not known, the proportional hazards model (PHM) can be carried out. The Cox proportional hazards model, compared to traditional analysis of variance (ANOVA) approaches and event-time models, has several advantages. These advantages are that when individuals are recorded at the tailrace, they can be explicitly included in the modelling, and the covariates may vary through time, therefore allowing passage hazards to change daily or seasonally.

However, this model also has its disadvantages. The main disadvantage is that hazards are calculated using the ranks of covariate values because the models are semi-parametric. For this reason, quantitative differences among treatments cannot be modelled as in traditional regression [18–20]. Therefore, hazard ratios compare the probability of the event occurring within a given time interval for an individual belonging to one group versus another. On the other hand, for continuous predictor variables, it compares for an increase in 1 unit of the variable.

The objective of this study is to investigate the socio-economic, demographic, and geographic predictors of under-five mortality in Ethiopia.

## Methods

The Demographic and Health Survey (DHS) is conducted in 2000, 2005, and 2011 in Ethiopia. This survey is a periodic, cross-sectional survey administered at the household level. For this study, data from the 2011 Ethiopian Demographic and Health Survey were used. The survey consisted of 624 selected enumeration areas (EAs). Complete household listings were carried out in each of the 624 EAs. For the survey, a sample of 17,817 households was selected. To estimate at the national level, all data of the survey were weighted and interviews were conducted with 9096 women aged 15–49 and 6033 men aged 15–59. Therefore, the 2011 EDHS sample was designed to provide estimates for the health and demographic variables of interest for Ethiopia as a whole: urban and rural areas of Ethiopia and 11 geographical areas [9–11].

The Cox proportional hazard regression model is useful for accessing the impact of the lifetime-related factors on the hazard function. These models play a significant role in the analysis of the lifetime data. In the model, the continuous random variable represents the lifetime of an individual (*t*), and the vector of explanatory variables related to (*X*), when *X* is given under the proportional hazard hypothesis. Therefore, let *x*_1_, *x*_2_ …, *x*_*p*_ be the values of *p* covariates *X*_1_, *X*_2_ …, *X*_*p*_. According to the Cox regression model, the hazard function is given as follows:

1 $$ h\left( t, X\right)={h}_0(t)\psi (X) $$

where *ψ*(*X*) = exp(∑_*i* = 1_^*p*^ *β*_*i*_ *x*_*i*_), *β* = (*β*_1_, *β*_2_, …, *β*_*p*_) is a 1 × *p* vector of regression parameters and *h*_0_(*t*) is the baseline hazard function at that time.

In the model, there are two unknown components, the regression parameter *β* and the baseline hazard function *h*_0_(*t*). The model component *h*_0_(*t*) is called the baseline hazard function.

The Cox regression model has a key assumption. The assumption is related to proportional hazards. The proportional hazards assumption states that the hazard ratio is constant over time or the hazard for an individual is proportional to the hazard for any other individual.

In its simplest form, the proportional hazard model can be given as

2 $$ {h}_i(t)={}_e{\displaystyle {\sum}_{i=1}^p{\beta}_i{X}_i{h}_0(t)} $$

where *h*_*i*_(*t*) is the hazard at time *t* of the *i* ^th^ individual and *h*_0_(*t*) is the baseline hazard at time *t. X*_*i*_ is a vector of covariate values corresponding to the *i*th individual, and *β* is a vector of coefficients to be estimated when the model is fit.

Let *X*_*i*_ = 0. Then, the hazard function of the *i* ^th^ individual is the baseline hazard function. Secondly, dividing both sides by *h*_0_(*t*) gives

$$ \frac{h_i(t)}{h_0(t)}={}_e{\displaystyle {\sum}_{i=1}^p{\beta}_i{X}_i}. $$

This equation shows where the term proportional comes from. In the equation, each individual, *e* ^*Xiβ*^ is constant across time. Furthermore, for every value of *t*, the *i* ^th^ individual’s hazard function is a constant proportion of the baseline hazard. Therefore, each individual’s hazard function is parallel to the *h*_0_(*t*). Moreover, the *i* ^th^ individual’s survival function is a constant power of the baseline survival function, i.e.,

$$ {S}_i\left( t, X\right)={\left[{S}_0(t)\right]}^{e^{X_i\beta}} $$

For proportional hazard function of *β*’s can be interpreted as time invariant shifters of the hazard function. Because of this property, the result can be interpreted as factors that affect risk, relative to the baseline risk where *S*_0_(*t*) is the essential life function *t* [20, 21].

The essential life function *t* can be written as

$$ {S}_0(t)={e}^{\left[-{\displaystyle {\int}_0^t{h}_0}(t) dt\right]}=\left[{H}_0(t)\right] $$

where *H*_0_(*t*) is the baseline cumulative hazard function.

The baseline hazard function includes a function of time. In the model, there may be time-dependent explanatory variables, which is any variable whose value can change in time. The value for a time-independent variable remains fixed [20, 22, 23].

Multivariate models with dependent random hazards provide a multivariate extension of the traditional univariate frailty model. This model allows taking mutual dependence of lifetimes into account in the analysis. For dependent lifetimes, survival models are useful. These models allow addressing more sophisticated questions about the nature of the mortality processes. To use Cox frailty model, assume conditional on the frailty, ***V***_*i*_ the hazard function *h*_*ik*_(*t*) for the failure time where *k* ^th^ (*k* = 1,2,…,*k*) children and *i* ^th^(*i* = 1,2,…,*n*) household follows proportional hazard from. Therefore, the model is given by

$$ {h}_{i k}(t)={h}_0(t){\boldsymbol{V}}_i\varPsi (t), t>0 $$

where ***V***_*i*_ is the grouped-level (household) frailty. The frailties in the model assumed unobservable unit mean and variance *θ* which is unknown. In each probabilistic sampling unit, which is kebele, households have different values of random effects. Here, *V*_*i*_ ' *s* reflect variability, and this shows heterogeneity of risks between households. From the model, the value of *o* frailty reflects that children from the same households are independent [24–26]. Therefore, the variance of the random effects lies between *o* and *α*. Therefore, large variance values indicate high heterogeneity between households. It also shows greater correlation between children with in the same households. Therefore, the frailty model follows the Gamma distribution and from the model correct *Z*-ratios expected [17, 20].

## Results

For the 2011 EDHS, the Cox regression analysis was fitted to the data to find factors affecting under-five mortality in Ethiopia. PROC PHREG in SAS 9.3 was used with additional options for model diagnosis like access option. The response variable for this analysis is child survival before the age of 5 and age at death. The covariate/predictors included in the model were social, economic, demographic, and geographic variables. These covariates are region, place of residence, religion, education, current age, mother’s age at first marriage, mother’s age at child’s birth, family size, relationship, husband’s education, working status, marital status, sex of child, pregnancy, time to collect water, source of drinking water, type of cooking facilities, toilet facilities, wealth index, smoking habits, main material of floor, roof, and walls. As well as the fixed effects, cluster/probability sampling unit (PSU) was considered as a random effect in the model.

The results of the Cox regression analysis with socio-economic, demographic, and geographic variables in the study are given in Table 1. From the results, it was found that region, type of residence, respondent’s current age, age of respondent at first birth, family size, age at first sex, total children ever born, and sex of child have significant effects on under-five mortality at the 5% of significance level. In addition to these main effects, the following two-way interaction effects were found. These effects were as follows: age of respondent at first birth and currently pregnant, education in single years and currently pregnant, number of household members and respondent currently working, total children ever born and current marital status, respondent’s current age and number of household members, age of respondent at first birth and number of household members, age of respondent at first birth and total number of children ever born, and number of household members and total number of children ever born.

**Table 1:** Type III tests for socio-economic, demographic, and geographic variables

Effect	DF	Chi-square	Pr > ChiSq
Region	10	32.0529	0.0004
Type of place of residence	1	4.5863	0.0322
Religion	5	6.222	0.2852
Education in single years	5	1.1057	0.9536
Respondent’s current age	1	206.2717	<0.0001
Age of respondent at 1st birth	1	81.9094	<0.0001
Family size	1	4.151	0.0416
Husband/partner’s education level	4	0.9479	0.9176
Current marital status	1	0.2692	0.6039
Age at 1st sex	1	5.8519	0.0156
Currently pregnant	1	0.9127	0.3394
Respondent currently working	1	2.9779	0.0844
Total children ever born	1	35.69	<0.0001
Sex of child	1	7.5047	0.0062
Time to get water	3	3.6774	0.2985
Source of drinking water	2	2.3263	0.3125
Type of cooking fuel	3	3.7077	0.2948
Type of toilet facility	2	2.3533	0.3083
Main floor material	2	3.6333	0.1626
Main roof material	3	0.3193	0.9564
Main wall material	2	0.3712	0.8306
Smoking	1	0.9341	0.3338
Age of respondent at 1st birth and currently pregnant	1	4.1467	0.0417
Education in single years and currently pregnant	1	4.6186	0.0316
Family size and respondent currently working	1	4.2749	0.0987
Total children ever born and current marital status	1	2.2638	0.0389
Respondent’s current age and family size	1	5.0619	0.0245
Age of respondent at 1st birth and family size	1	8.4761	0.0036
Age of respondent at 1st birth and total children ever born	1	7.0545	0.0079
Family size and total children ever born	1	14.6694	0.0001

**Fig. 1:**
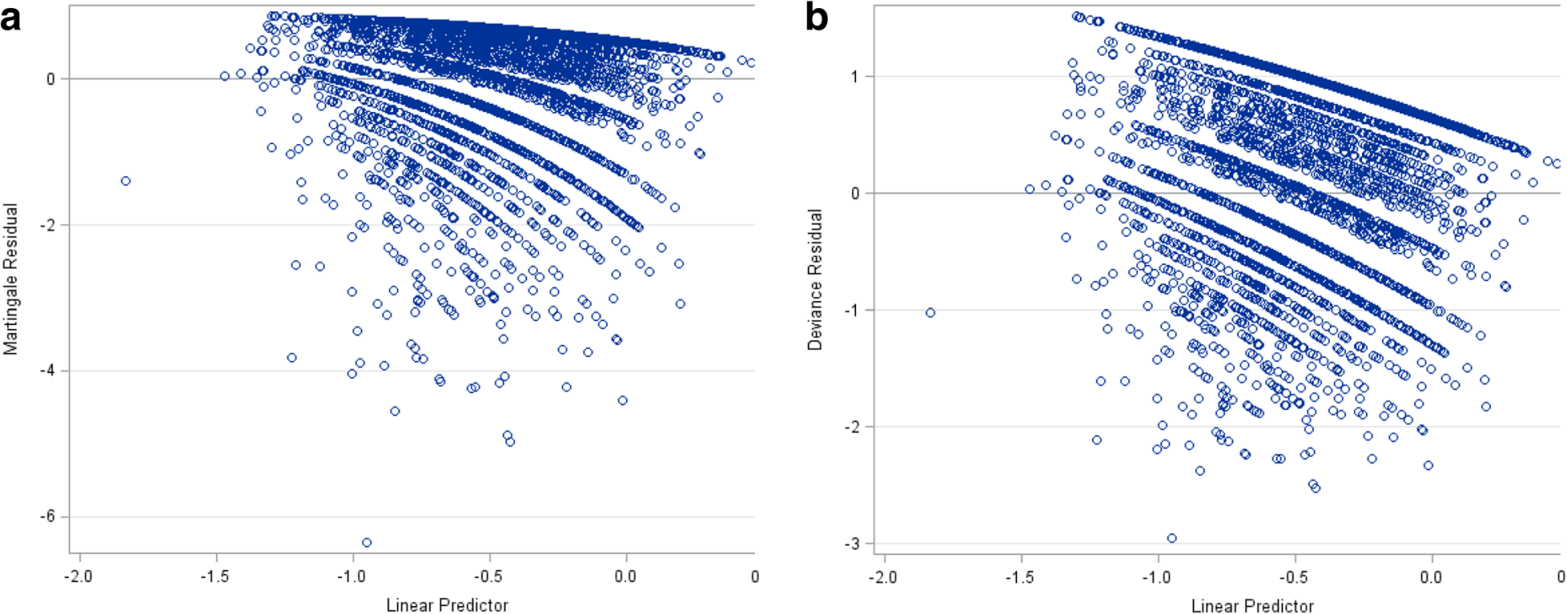
**a** Martingale residual plot and **b** deviance residual plot

The resulting plots for residual assessment are shown in Fig. 1. The Martingale residuals are skewed because of the single event setting of the Cox model. The Martingale residual plot shows an isolation point, but this observation is no longer distinguishable in the deviance residual plot. Therefore, there is no indication of a lack of fit of the model to individual observations.

**Fig. 2:**
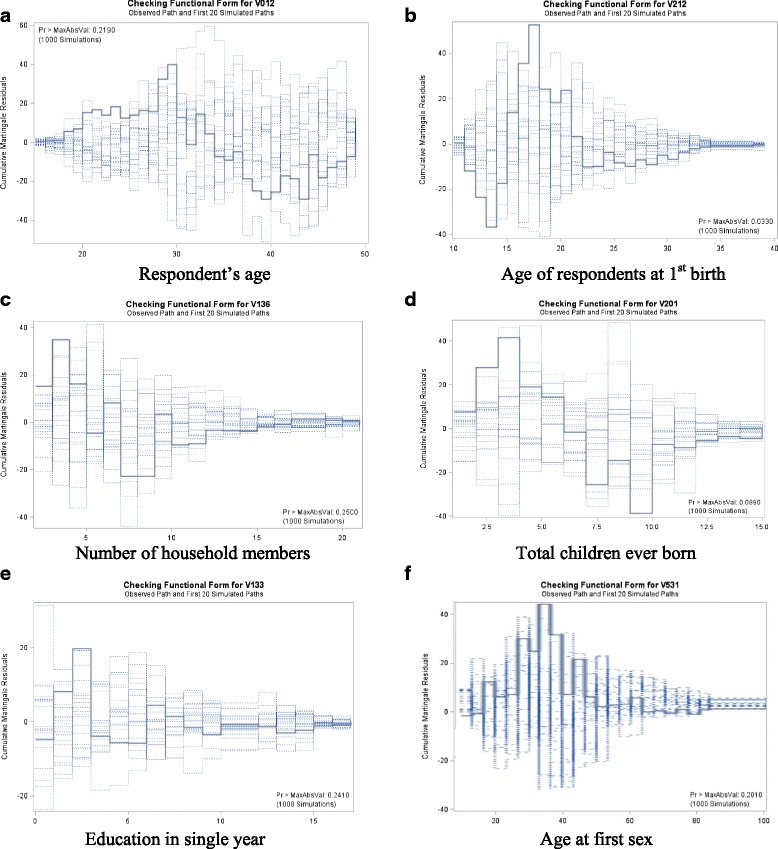
**a**–**f** Cumulative Martingale residuals for age, age of respondents at first birth, number of household members, total children ever born, education in single year, and age at first sex

The plot in Fig. 2 displays the observed cumulative Martingale residual process for the respondent’s age, age of respondents at first birth, number of household members, total number of children ever born, education in single year, and age at first sex, together with simulated realizations from the null distribution. These plots are displayed using option ASSESS statement using SAS. In the plot, the observed process is compared to the simulated realizations. The curves of observed cumulative Martingale residuals presented in Fig. 2 indicate that age, age of respondents at first birth, number of household members, total children ever born, education in single year, and age at first sex are appropriate terms in the model. The Kolmogorov-type supremum test results for all the covariates are shown in Table 2. From the result, it can be seen that the proportional hazards assumption appears to be satisfied.

**Table 2:** Supremum test for proportional hazards assumption

Variable	Maximum absolute value	Replications	Seed	*p* value
Respondent’s current age	39.9485	1000	7548	0.219
Education in single years	19.7343	1000	7548	0.241
Number of household members	34.8539	1000	7548	0.250
Total children ever born	41.6613	1000	7548	0.089
Age of respondent at 1st birth	52.6433	1000	7548	0.033
Age at 1st sex	43.7919	1000	7548	0.201

Table 3 shows the coefficient estimates and associated statistics for fixed effects and the variance of frailty (cluster/probability sampling unit (PSU)). As can be seen from the table, the frailty value is significantly greater than zero (*θ* = 1.721; *p* value = 0.004). Therefore, this result shows that there are factors that have an effect on the hazard of death. From the result, it was obvious that the region variable was important (*p* = 0.0004). Moreover, the gamma frailty with value 1.721 shows that there are larger unmeasured household effects present in the model. The 2011 EDHS under-five children who lived in Addis Ababa had a lower hazard (risk) of death than those in the Tigray region. The estimated hazard ratio was 0.681 indicating that under-five children who lived in Addis Ababa had a 32% lower hazard (risk) of death than the under-five children who lived in the Tigray region. Similarly, under-five children who lived in the Somali region had a lower hazard (risk) of death compared to those who lived in the Tigray region, followed by Afar, Benishangul-Gumuz, Amhara, and Gambella regions. Furthermore, under-five children who lived in rural areas had a higher hazard (risk) of death compared to those living in urban areas. The estimated hazard ratio was 1.180, indicating that under-five children who lived in rural areas had 18% more hazard (risk) of death than those living in urban areas.

**Table 3:** Estimates from the CPH on the effect of socio-economic, demographic and geographic variables on under-five mortality

Parameters	Estimates	Hazard ratio	CI		*p* value
			Lower	Upper	
Region (Ref. Tigray)					
Addis Ababa	−0.383	0.681	0.421	0.796	0.048
Affar	−0.296	0.744	0.564	0.841	0.001
Amhara	−0.04	0.961	0.285	1.087	0.539
Benishangul-Gumuz	−0.258	0.772	0.586	0.881	0.001
Dire Dawa	−0.192	0.825	0.645	0.981	0.044
Gambela	−0.067	0.936	0.458	1.179	0.465
Harari	0.007	1.007	0.047	1.085	0.947
Oromiya	−0.178	0.837	0.687	0.965	0.014
SNNP	−0.239	0.787	0.649	0.857	0.002
Somali	−0.377	0.686	0.611	0.749	<0.0001
Type of place of residence (Ref. urban)					
Rural	0.165	1.18	1.085	1.254	0.01
Currently pregnant (Ref. no)					
Yes	−0.145	0.865	0.805	0.930	<0.0001
Respondent’s current age	−0.051	0.95	0.897	0.967	<0.0001
Age of respondent at 1st birth	0.05	1.051	1.012	1.154	<0.0001
Family size	−0.019	0.981	0.785	0.997	0.022
Age at 1st sex	0.012	1.012	1.004	1.124	0.004
Total children ever born	0.065	1.067	1.011	1.189	<0.0001
Sex of child					
Female	−0.089	0.915	0.759	0.995	0.002
Age of respondent at 1st birth and currently pregnant (Ref. yes)					
No/do not know	−0.028	0.972	0.743	0.987	0.045
Education in single years and currently pregnant (Ref. yes)					
No/do not know	−0.254	0.776	0.574	0.857	0.0013
Family size and respondent currently working (Ref. yes)					
No	0.1105	1.117	1.087	1.204	0.0031
Respondent’s current age and family size	−0.005	0.995	0.847	0.998	0.0118
Age of respondent at 1st birth and family size	0.007	1.007	1.002	1.106	0.0105
Age of respondent at 1st birth and total children ever born	−0.003	0.997	0.785	0.999	0.0439
Family size and total children ever born	0.025	1.025	1.005	1.158	<0.0001
Frailty (cluster or PSU)	1.721		1.487	1.983	0.004

The proportional hazards regression model for these data with age at first sex as the predictor is found to be 1.012 with C.I. (1.004, 1.124). Therefore, with each yearly increase in age of a mother at first sex, the risk of her child dying before reaching the age of 5 is increased by 1.2%.

In addition to the main effects, there were interaction effects which have an influence on the under-five mortality of Ethiopia. Among these effects, the first one is the interaction between the age of the respondent at first birth and whether currently pregnant. The result is presented in Table 3. From the table, it can be seen that as the age of respondents at first birth increases, the risk of the child dying before the age of 5 decreases, for women who are not pregnant (HR = 0.972, C.I. (0.743, 0.987)). But the risk of a child dying before the age of 5 is lower for women who were not pregnant.

The other interaction effect is between education in single years and whether currently pregnant. The result is presented in Table 3. From the table, it can be seen that as the respondents’ educational level increases, the risk of the child dying before the age of 5 decreases, for both not pregnant women (HR = 0.776, C.I. (0.574, 0.857)). As with the interaction between the age of the respondent at first birth and whether currently pregnant, the risk of a child dying before the age of 5 was lower for women who were not pregnant.

The interaction between family size and respondent’s current work status was found to be significant. The result is presented in Table 3. The result indicates that the risk for a child to not reach the age of 5 increases as family size increases for unemployed women. Moreover, for respondents who are not working, the result is higher than for respondents who are working (HR = 1.117, C.I. (1.087, 1.204)).

The joint effect of a respondent’s current age and family size was one of the interaction effects (Table 1). As the result indicates, the chance of a child dying before reaching age 5 increases as the age of the respondent increases for an increase in family size. Moreover, the chance of a child dying before reaching the age of 5 is highest for maximum family size, followed by median family size and lastly minimum family size.

The effect of the interaction between age of respondent at first birth and family size was found to be significant (Table 1). Unlike the interaction effect between current age and family size, the interaction between age of respondent at first birth and family size shows increase. Therefore, for an increase in respondents age at first birth and family size, the risk of a child to reach age 5 increases by 7% (HR = 1.007, C.I. (1.002, 1.106)). The other two-way significant interaction effects are between age of respondent at first birth and total children ever born and between family size and total children ever born. This result is presented in Table 3.

**Fig. 3:**
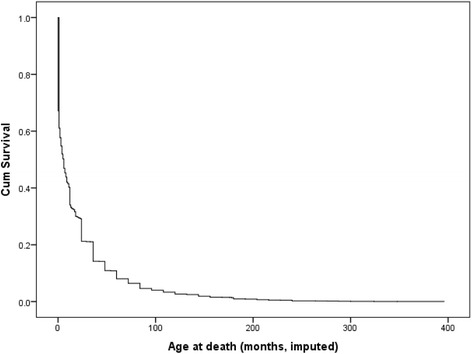
Survival function at mean of covariates

The survival function at mean of covariates is given in Fig. 3. From the figure, the survival curves give a visual representation of the life tables. The horizontal axis shows the time to event. The vertical axis shows the probability of surviving or the proportion of people surviving. At time 0, the survival probability is 1.0 (or 100% of the participants are alive). Moreover, as the age of a child increases, the probability of the child surviving decreases.

## Discussion and conclusions

The risk of a child dying before reaching 5 years of age is highest in sub-Saharan African countries. The under-five mortality rate is greater than 100 deaths per 1000 live births [3, 4], which is about eight times higher than that in the WHO European Region where the mortality rate is 12 deaths per 1000 live births [27, 28]. In addition, large inequities exist between high-income and low-income countries regarding the deaths of under 5-year-olds. In 2012, the under-five age mortality rate in low-income countries was 82 deaths per 1000 live births, more than 13 times the average rate in high-income countries (6 deaths per 1000 live births). Reducing these inequities across countries and saving more children’s lives by ending preventable child deaths are important priorities [29, 30].

Under-five mortality rates have shown a substantial decline in Ethiopia. This has been achieved since the 1960s implementation of health interventions [5]. Mortality trends can be studied by associating mortality rates for the three 5-year periods for a single survey or from several surveys over time. The recently conducted 2011 Ethiopian Demographic and Health Survey results show a decline in all levels of childhood mortality. Infant mortality has declined by 42% over the 15-year period preceding the survey, from 101 deaths per 1000 live births to 59 deaths per 1000 live births. Similarly, under-five mortality also shows a decline by 47% over the same period, from 166 deaths per 1000 live births to 88 deaths per 1000 live births. Based on the three DHS surveys (2000, 2005, and 2011), mortality trends can also be examined. According to these surveys, infant and under-five mortality rates show a continuous declining trend. Under-five mortalities decreased from 166 deaths per 1000 live births in the 2000 survey to 88 in 2011, while infant mortality decreased from 97 deaths per 1000 live births in the 2000 survey to 59 in the 2011 survey. Furthermore, crude death rates also show substantial declines over the past 10 years [9–12]. As the record shows, infant and under-five mortalities in Ethiopia have continued to decline over the past 25 years. But, in the last 10 years, there has been a more pronounced reduction [11, 31]. Recent study conducted by Ayele, Zewotir, and Mwambi in 2015 supported the reduction of under-five mortality [32]. However, despite these decreases, mortality rates are still high in Ethiopia. Therefore, to identify the socio-economic and demographic factors influencing under-five mortality, survival analysis has been used.

For this study, the nationally representative data from the 2011 Ethiopian Demographic and Health Survey was used. The objective was to investigate the socio-economic, demographic, and geographic predictors of under-five mortality in Ethiopia. The Cox regression analysis technique has been applied to identify the important socio-economic, demographic, and geographic predictors of under-five mortality. A Cox regression model is used when the relative risk values for different levels of variables measured at different levels of socio-economic, demographic, and geographic variables. Even though infant and under-five mortality rates have been decreasing in many parts of sub-Saharan Africa in recent decades, they remain among the highest in the world. In the literature, the decline in mortality is well documented, but it has been difficult to determine the various socio-economic, demographic, and geographic factors associated with this decline.

Therefore, for this study, the Cox regression model was used as it is a procedure which is useful for modelling the time to a specified event, based upon the values of given covariates. For this study, 23 covariates were used to predict the status of under-five mortality. Moreover, the death of a child (status variable) is the dependent variable in Cox regression and is a binary variable. Time variable (age at death) measures the duration to the event defined by the status variable. The covariates (independent variables) for this study contain both the categorical and continuous variables.

The results of this model which was applied to under-five mortality in Ethiopia showed that under some conditions, socio-economic, demographic, and social factors all have a great effect on a child’s survival up to age 5. Additional to this, the Cox regression model determined that the data obtained during the 2011 Ethiopian Demographic and Health Survey is of great importance. From the results of this present study, it can be concluded that children under-five, who live in Addis Ababa, have a lower hazard (risk) of death. Moreover, for older mothers, the chances of the child dying before reaching age 5 are low. In addition, with each year’s increase in the age of a mother at first sex, the risk of a child dying before reaching the age of 5 decreases. For mothers who gave birth at an older age, and for educated mothers, and for women who are not pregnant, the chances of a child reaching age 5 is higher. Moreover, for large households and for mothers who are not working, the chances of a child not reaching age 5 is higher. Therefore, by giving birth at an older age, and not being pregnant again before the child reaches age 5, is one means of reducing under-five mortality.
